# Effects of Breast Cancer Adjuvant Chemotherapy Regimens on Expression of the Aging Biomarker, *p16^INK4a^*

**DOI:** 10.1093/jncics/pkaa082

**Published:** 2020-12-18

**Authors:** Shlomit S Shachar, Allison M Deal, Katherine E Reeder-Hayes, Kirsten A Nyrop, Natalia Mitin, Carey K Anders, Lisa A Carey, E Claire Dees, Trevor A Jolly, Gretchen G Kimmick, Meghan S Karuturi, Raquel E Reinbolt, JoEllen C Speca, Hyman B Muss

**Affiliations:** 1 Rambam Health Campus, School of Medicine, Haifa, Israel; 2 Lineberger Comprehensive Cancer Center, University of North Carolina at Chapel Hill, Chapel Hill, NC, USA; 3 School of Medicine, University of North Carolina at Chapel Hill, Chapel Hill, NC, USA; 4 Sapere Bio, Research Triangle Park, NC, USA; 5 School of Medicine, Duke University, Durham, NC, USA; 6 MD Anderson Cancer Center, University of Texas, Houston, TX, USA; 7 Comprehensive Cancer Center, Ohio State University, Columbus, OH, USA; 8 UNC Rex Healthcare, Raleigh, NC, USA

## Abstract

**Background:**

Although chemotherapy saves lives, increasing evidence shows that chemotherapy accelerates aging. We previously demonstrated that mRNA expression of *p16^INK4a^*, a biomarker of senescence and molecular aging, increased early and dramatically after beginning adjuvant anthracycline-based regimens in early stage breast cancer patients. Here, we determined if changes in *p16^INK4a^* expression vary by chemotherapy regimen among early stage breast cancer patients.

**Methods:**

We conducted a study of stage I-III breast cancer patients receiving adjuvant or neoadjuvant chemotherapy. *p16^INK4a^* expression was analyzed prechemotherapy and postchemotherapy (median 6.2 months after the last chemotherapy) in peripheral blood T lymphocytes. Chemotherapy-induced change in *p16^INK4a^* expression was compared among regimens. All statistical tests were 2-sided.

**Results:**

In 146 women, chemotherapy was associated with a statistically significant increase in *p16^INK4a^* expression (accelerated aging of 17 years; *P* < .001). Anthracycline-based regimens were associated with the largest increases (accelerated aging of 23 to 26 years; *P* ≤ .008). Nonanthracycline-based regimens demonstrated a much smaller increase (accelerated aging of 9 to 11 years; *P* ≤ .15). In addition to the type of chemotherapy regimen, baseline *p16^INK4a^* levels, but not chronologic age or race, were also associated with the magnitude of increases in *p16^INK4a^*. Patients with lower *p16^INK4a^* levels at baseline were more likely to experience larger increases.

**Conclusions:**

Our findings suggest that the aging effects of chemotherapy may be influenced by both chemotherapy type and the patient’s baseline *p16^INK4a^* level. Measurement of *p16^INK4a^* expression is not currently available in the clinic, but nonanthracycline regimens offering similar efficacy as anthracycline regimens might be favored.

There is increasing evidence that cancer treatment, especially chemotherapy, accelerates human aging (1-4). The most compelling evidence comes from childhood cancer survivorship studies that found that by age 50 years the cumulative incidence of at least 1 severe, disabling, or life-threatening condition was 53.6%. In addition, 30% of survivors who reached age 35 years without any severe or life-threatening condition developed a life-threatening condition within 10 years (5). Accumulation of chronic diseases due to accelerated aging led to an 8.4-fold increased risk of death compared with the general population.

Senescence is a cellular mechanism that is activated in response to stress and DNA damage and promotes permanent inhibition of the cell cycle of damaged cells. This targeted control of the cell cycle serves as a protective mechanism essential for minimizing the proliferation of damaged cells that can lead to tumor initiation and growth (6). Over time (or after repeated damage), an accumulation of senescent cells in tissues contributes to perturbations in the ability of tissues to undergo repair, in part because of the decline in the replicative function of self-renewing stem cells and an increase in local inflammation (7-10). Therefore, although senescent cells protect tissues from becoming cancerous, the accumulation of senescent cells promotes chronic inflammation, declines in organ function, and acceleration of age-related diseases (eg, atherosclerosis, idiopathic lung fibrosis, osteoarthritis, bone loss, hepatic steatosis, and cognitive decline) (11).

Finding biomarkers of aging that capture changes over the entire lifespan and reflect the clinical aspects of aging is a rich and expanding area of research (12,13). Potential biomarkers include inflammatory markers, telomere length (14-18), sarcopenia, DNA methylation (19,20), and maximal oxygen consumption (vO^2^max) (21). Expression of *p16^INK4a^*, a biomarker of senescence, is considered a fundamental and dynamic measurement of molecular aging with a 10-fold increase in gene expression between ages 20 and 80 years (22). *p16^INK4a^* is a tumor suppressor molecule that acts to arrest cell proliferation, resulting in multi-organ cell senescence in murine models (23). Maintenance of the *p16^INK4a^* transcript is required for maintenance of the senescent phenotype, and senescent cells remain in tissues indefinitely (24-27).

In humans, *p16^INK4a^* expression can be readily measured in T cells and is a robust marker of age in normal controls (28). Expression of *p16^INK4a^* in human T cells is influenced by a variety of age-promoting stimuli, including cigarette smoking, physical inactivity (28), cytotoxic chemotherapy administration (29), chronic HIV infection (30), and bone marrow transplantation (4). We previously reported rapid and persistent increases in *p16^INK4a^* mRNA expression in the peripheral blood T cells in a small cohort of women receiving anthracycline-based adjuvant chemotherapy for early stage breast cancer, whereas in a second cohort of early stage breast cancer patients who did not receive chemotherapy, *p16^INK4a^* expression levels were similar to healthy volunteers (29).

Our initial study of chemotherapy-induced aging was done in early stage breast cancer patients, almost all of whom received anthracycline-based regimens (29). In the current study, we postulated that currently used chemotherapy regimens containing agents with different mechanisms of action would differ in their propensity to induce senescence in T cells. To address this question, we evaluated if chemotherapy-induced changes in *p16^INK4a^* mRNA expression differed among 4 commonly used adjuvant chemotherapy regimens. We also examined the contribution of different patient and clinical characteristics, including chronologic age and baseline *p16^INK4a^* expression, in predicting the magnitude of increased *p16^INK4a^* expression in response to chemotherapy treatment.

## Methods

### Study Participants

Patients were recruited between April 2011 and August 2018 from university-affiliated hospitals. Women aged 21 years or older with histologically confirmed stage I-III breast cancer and scheduled for adjuvant or neoadjuvant chemotherapy were offered participation in 3 Lineberger Comprehensive Cancer Center (LCCC) studies: LCCC1027 (NCT01305954), LCCC1334 (NCT02167932), and LCCC1410 (NCT02328313). The protocols differed in age criteria for inclusion (LCCC1334 for women aged 21 to 64 years, LCCC1410 for women aged 65 years or older, LCCC1027 for women aged 18 years or older). All studies were approved by the University of North Carolina at Chapel Hill (UNC) LCCC protocol review committee and the institutional review boards of each study site. Written informed consent meeting all university and federal guidelines were obtained from each participant. A total of 19, 75, and 52 participants were enrolled in LCCC1027, LCCC1334, and LCCC1410, respectively.

### Chemotherapy Regimens

Patients enrolled in these studies received chemotherapy as determined by their oncologist based on tumor stage and breast cancer phenotype as suggested by National Comprehensive Cancer Network guidelines (31). For statistical analysis, regimens were arranged into 4 groups: 1) doxorubicin, cyclophosphamide, and paclitaxel (AC-T); 2) docetaxel and cyclophosphamide ± anti-HER2 therapy (TC); 3) docetaxel and carboplatin + anti-HER2 therapy (TCH); and 4) doxorubicin and cyclophosphamide plus paclitaxel and carboplatin (AC-TC). Granulocyte colony-stimulating factors (pegfilgrastim) were routinely used in patients receiving docetaxel and cyclophosphamide, docetaxel and carboplatin, and doxorubicin and cyclophosphamide (when administrated every 2 weeks—“dose dense”). For patients on chemotherapy regimens that included anti-HER2 therapy for 12 months, postchemotherapy blood draws were done while patients were still receiving anti-HER2 therapy.

### *p16^INK4a^* Assay

Blood samples were drawn prior to chemotherapy initiation and at least 60 days after the completion of chemotherapy. The median time from the last chemotherapy treatment to the postchemotherapy blood sample was 6.2 months (range = 2.1-14.3). Blood was drawn into lavender-top EDTA tubes, T cells were isolated, and expression of *p16^INK4a^* mRNA in peripheral blood T lymphocytes was determined using TaqMan real-time quantitative reverse transcription polymerase chain reaction. Expression analysis was performed by Sapere Bio (formerly HealthSpan Dx), using technology described previously (29). Every run included external and internal controls to monitor assay performance. Cycle threshold values over 37 were considered below detection and excluded from the analysis. The overall precision (reproducibility based on run-to-run and between operators’ analytical validation) of *p16^INK4a^* measurement was 0.8 Ct. The same assay and quality control procedures were used for all samples.

### Statistical Analysis

Descriptive statistics are provided to summarize the study sample. Differences in age by regimen were compared using ANOVA, and paired *t* tests evaluated changes in *p16^INK4a^* expression overall and within each regimen. Linear regression was used to model the relationship between patient characteristics with the change in *p16^INK4a^* expression. Fisher exact test compared the rates of *p16^INK4a^* expression change by regimen. All analyses were conducted using SAS statistical software v9.4 (Cary, NC). Conversion of change in log2 *p16* to years of chronologic aging was calculated using the formula: Δlog2p16/0.028 derived from a 633-patient cohort (32). All statistical tests were 2-sided, and a *P* value of less than .05 was considered statistically significant.

## Results

### Patient Characteristics

We enrolled 146 patients diagnosed with early stage breast cancer who received neoadjuvant or adjuvant chemotherapy. Their baseline characteristics are presented in Table 1. The mean age was 57 years (range = 28-81). Karnofsky Performance Status was greater than 80% in 92% of patients. African American patients comprised 15.8% of the cohort, 50.7% had hormone receptor-positive and HER2-negative tumors, and 62.3% were treated with adjuvant chemotherapy. Nearly half the patients (47.9%) were treated with anthracycline-based regimens (AC-T or AC-TC), 34.9% with docetaxel and cyclophosphamide (TC), and 17.1% with docetaxel and carboplatin + anti-HER2-directed therapy (TCH). Radiation therapy was administered to 73% of patients after the completion of chemotherapy. As shown in Table 2, patient characteristics were closely associated with the different chemotherapy regimens. For example and as expected, patients with higher stage cancers (stage II and III) were more likely to receive anthracycline (AC)–containing chemotherapy, whereas TC was more likely administered in women with estrogen receptor–positive tumors and lower stage tumors; the majority of women receiving AC-TC had triple-negative breast cancer.

**Table 1.** Patient characteristics

Variable	All patients
	(n = 146)
Overall age at consent, y	
Mean (SD)	57 (12.2)
Range, y	28-81
Race, No. (%)	
White	110 (75.3)
African American	23 (15.8)
Other	13 (8.9)
Breast cancer stage, No. (%)^a^	
I	32 (21.9)
II	81 (55.5)
III	33 (22.6)
Phenotype, No. (%)	
Hormone receptor–positive and HER2-negative	74 (50.7)
Hormone receptor–negative and HER2-negative (triple negative)	42 (28.8)
HER2-positive and hormone receptor–negative	14 (9.6)
HER2-positive and hormone receptor–positive	16 (11.0)
Timing of chemotherapy, No. (%)	
Neoadjuvant	54 (37.0)
Adjuvant	91 (62.3)
Both	1 (0.7)
Treatment regimens, No. (%)	
AC-T (doxorubicin and cyclophosphamide then paclitaxel and carboplatin)^b^	53 (36.3)
AC-TC (doxorubicin and cyclophosphamide then paclitaxel and carboplatin)^c^	17 (11.6)
TC (docetaxel and cyclophosphamide)^d^	51 (34.9)
TCH (docetaxel and carboplatin + anti-HER2 therapy)^e^	25 (17.1)

a American Joint Committee on Cancer stage version 7.

b Nine patients had paclitaxel before doxorubicin and cyclophosphamide. Doxorubicin and cyclophosphamide given every 2 weeks with growth factors (5 patients got anthracycline every 3 weeks); 3 patients received anti-HER2 therapy with AC-T.

c TC given first in 11 patients.

d Given every 3 weeks with growth factors; 2 patients received anti-HER2 therapy with TC.

e Given every 3 weeks with growth factors; all received trastuzumab, and 19 patients received pertuzumab in addition to trastuzumab.

**Table 2.** Patient characteristics by chemotherapy regimen

Variable	Regimen					
	AC-T	AC-TC	TC	TCH	*P* ^a^	*P*
	(n = 53)	(n = 17)	(n = 51)	(n = 25)		AC vs non-AC^a^
Chronological age, y						
Mean (SD)	55.7 (11.5)	48.9 (12.5)	60.5 (11.3)	58.0 (13.1)	.005	.006
Race, No. (%)						
African American	12 (22.6)	2 (11.8)	4 (7.8)	5 (20.0)	.48	.35
White	37 (69.8)	14 (82.3)	42(82.4)	17 (68.0)		
Other	4 (7.5)	1 (5.9)	5 (9.8)	3 (12.0)		
Phenotype, No. (%)						
Estrogen receptor–positive	36 (67.9)	1 (5.9)	39 (76.5)	14 (56.0)	<.001	.04
Estrogen receptor–negative	17 (32.1)	16 (94.1)	12 (23.5)	11 (44.0)		
Stage, No. (%)						
I	7 (13.2)	1 (5.9)	22 (43.1)	2 (8.0)	<.001	<.001
II	27 (50.9)	10 (58.8)	29 (56.9)	15 (15.0)		
III	19 (35.8)	6 (35.3)	0 (0)	8 (32.0)		
Timing of chemotherapy, No. (%)						
Adjuvant	29 (54.7)	2 (11.8)	49 (96.1)	11 (44.0)	<.001	<.001
Neoadjuvant	23 (43.4)	15 (88.2)	2 (3.9)	14 (56.0)		
Both	1 (1.9)	0 (0)	0 (0)	0 (0)		
Radiation postchemotherapy, No. (%)						
Yes	40 (75.5)	13 (76.5)	35 (68.6)	18 (72.0)	.73	.29
No	13 (24.5)	4 (23.5)	16 (31.4)	7(28.0)		

a ANOVA for age (continuous variable) and Fisher exact test for all categorical variables. All tests 2-sided. AC-T = doxorubicin and cyclophosphamide then paclitaxel and carboplatin; AC-TC = doxorubicin and cyclophosphamide then paclitaxel and carboplatin; TC = docetaxel and cyclophosphamide; TCH = docetaxel and carboplatin + anti-HER2 therapy.

### Change in *p16^INK4a^* Expression From Baseline to Posttreatment

Mean differences in *p16^INK4a^* expression from baseline to the follow-up visit are presented in Table 3. Overall, chemotherapy regimens were associated with a 1.4-fold increase in *p16^INK4a^* expression (equivalent to a 17-year acceleration in aging; *P* < .001). Anthracycline-based regimens (AC-T or AC-TC) were associated with the largest mean change in *p16^INK4a^* expression (1.6- to 1.7-fold increase; equivalent to a 23- to 26-year acceleration in aging; *P* ≤ .008), whereas smaller mean changes in *p16^INK4a^* expression were observed in patients receiving nonanthracycline chemotherapy regimens: TCH (equivalent to a 9-year acceleration in aging; *P* = .03) or TC (equivalent to 11-year acceleration in aging; *P* = .15).

**Table 3.** Chemotherapy-induced change from baseline in *p16^INK4a^* mRNA expression to the follow-up visit by chemotherapy regimen and overall

Regimen	Time point	No.	Mean log2 *p16^INK4a^* (95% CI)	Mean log2 change in *p16^INK4a^* (95% CI)	*P* ^a^	Absolute change in *p16^INK4^* (95% CI)	Change in age (95% CI), y
Overall	Baseline	146	9.62 (9.46 to 9.77)	0.47 (0.33 to 0.61)	<.001	1.39 (1.25 to 1.54)	17 (12 to 22)
	Follow-up visit	146	10.09 (9.93 to 10.24)				
AC-T (Dose-dense doxorubicin and cyclophosphamide followed by paclitaxel)	Baseline	53	9.53 (9.28 to 9.78)	0.65 (0.46 to 0.84)	<.001	1.57 (1.38 to 1.79)	23 (16 to 30)
	Follow-up visit	53	10.18 (9.93 to 10.43)				
AC-TC (Doxorubicin and cyclophosphamide followed by paclitaxel and carboplatin)	Baseline	17	9.34 (8.87 to 9.82	0.74 (0.22 to 1.25)	.008	1.67 (1.16 to 2.38)	26 (8 to 45)
	Follow-up visit	17	10.08 (9.65 to 10.52)				
TC (Docetaxel and cyclophosphamide ± anti-HER2 therapy)	Baseline	51	9.79 (9.52 to 10.05)	0.30 (0.04 to 0.57)	.03	1.23 (1.03 to 1.48)	11 (1 to 20)
	Follow-up visit	51	10.09 (9.78 to 10.40)				
TCH (Docetaxel and carboplatin + anti-HER2 therapy)	Baseline	25	9.64 (9.25 to 10.02)	0.26 (−0.10 to 0.63)	.15	1.20 (1.00 to 1.55)	9 (−4 to 23)
	Follow-up visit	25	9.90 (9.57 to 10.23)				

a Paired *t* test and all 2-sided. CI = confidence interval.

### Baseline *p16^INK4a^* and Change in *p16^INK4a^* Expression

**Figure 1.**
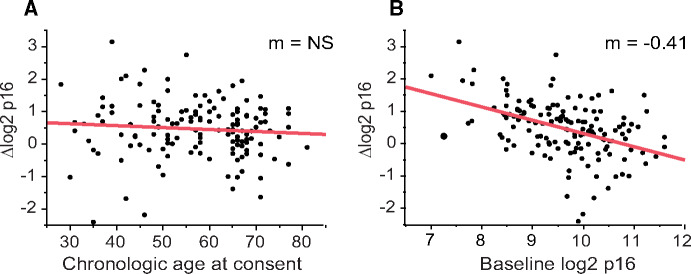
Chemotherapy-induced changes in *p16^INK4a^* mRNA expression correlate with baseline *p16^INK4a^* expression but not chronologic age. Correlation between chronologic age **(A)** or baseline *p16^INK4a^* expression **(B)** and chemotherapy-induced change in *p16^INK4a^* expression. AC = anthracycline; M = regression slope; non-AC = nonanthracycline-containing; NS = not statistically significant (*P* = .32).

**Figure 2.**
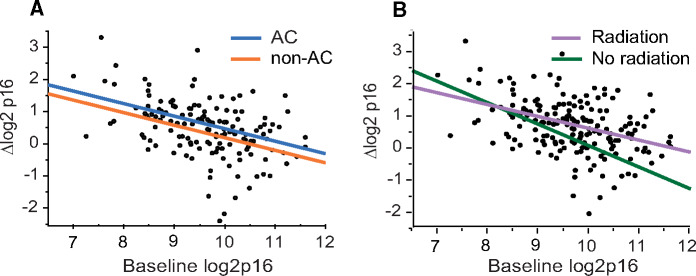
Patient’s expression levels of *p16^INK4a^* prior to chemotherapy are a major predictor of chemotherapy-induced change in *p16^INK4a^* mRNA expression. Correlation between baseline *p16^INK4a^* expression and chemotherapy-induced change in *p16^INK4a^* expression in patients receiving anthracycline (**blue line**) or nonanthracycline-containing regimens (**orange line**) **(A)** or patients who received (**lavender line**) or did not received (**green line**) postchemotherapy radiation **(B)**. AC = anthracycline.

Having established that the increase in *p16^INK4a^* expression may be driven by the type of chemotherapy regimen, we tested if patient characteristics contributed to the magnitude of chemotherapy-induced increase in *p16^INK4a^* expression. Surprisingly, we found that baseline *p16^INK4a^* expression, and not chronologic age, correlated with the magnitude of chemotherapy-induced increase in *p16^INK4a^* expression (Figure 1). When we separated the cohort into patients receiving anthracycline- vs nonanthracycline-based chemotherapy, the regression slopes of association between baseline *p16^INK4a^* expression and chemotherapy-induced change in *p16^INK4a^* expression were parallel between treatment groups (Figure 2, A). These findings suggest that although anthracycline-containing chemotherapy regimens cause larger increases in *p16^INK4a^* expression levels than nonanthracycline regimens, the propensity to increase *p16^INK4a^* expression was dependent on the baseline *p16^INK4a^* expression and not an individual regimen.

Breast or locoregional radiation in this cohort was given to 73% of patients. There was no statistically significant difference in the anthracycline chemotherapy–induced increase in *p16^INK4a^* expression compared with those who did not receive radiation therapy (*P* = .29). As shown in Figure 2, B, regardless of whether patients received radiation or not, a similar trend was observed for an inverse correlation between *p16^INK4a^* expression levels at baseline, and the magnitude of chemotherapy-induced change in *p16^INK4a^* expression. A more detailed quantitative analysis of the impact of radiation on the chemotherapy-induced increase in *p16^INK4a^* expression was not possible, because there was a statistical interaction between baseline *p16^INK4a^* expression and receipt of radiation (p16*radiation, *P* = .06). This finding is not surprising because the decision to administer radiation is based on the type or surgery (partial or total mastectomy) and stage.

To understand the effect of baseline *p16^INK4a^* expression, chemotherapy regimen, and race on the chemotherapy-induced increase in *p16^INK4a^* expression, we analyzed these variables using univariate and multivariate regression analyses. As shown in Table 4, in a multivariate analysis, both baseline *p16^INK4a^* expression and chemotherapy regimen, but not race, were statistically significant predictors of a chemotherapy-induced increase in *p16^INK4a^* expression. These findings suggest that patients with lower baseline levels of *p16^INK4a^* expression are more likely to experience larger chemotherapy-induced increases in *p16^INK4a^* expression.

**Table 4.** Linear regression parameter estimates for the magnitude of change in *p16^INK4a^* mRNA expression

Variable	Univariate		Multivariable^a^	
	Estimate	*P* ^b^	Estimate	*P* ^b^
Chronologic age at consent	−0.006	.31	0.010	.09
Baseline *p16^INK4a^* mRNA expression	−0.410	<.001	−0.435	<.001
Regimen, AC vs non-AC	0.380	.008	0.325	.01
Race, African American vs all others	0.224	.26	NS	NS

a Regimen-adjusted analysis was based on AC- vs non-AC–containing regimens. AC = anthracycline.

b Linear regression and 2-sided.

**Figure 3.**
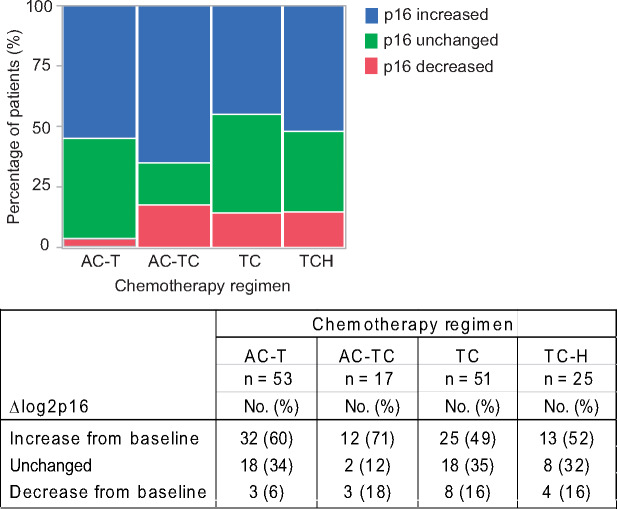
Patients whose *p16^INK4a^* expression levels are increased by chemotherapy are not restricted to any 1 treatment. Distribution of patients whose *p16^INK4a^* levels increased above assay precision is similar among chemotherapy regimens commonly used to treat early stage breast cancer patients.

Finally, to better understand how these data might apply to a clinical setting, we evaluated whether specific chemotherapy regimens differed in their propensity to increase *p16^INK4a^* expression (Figure 3). Changes in *p16^INK4a^* expression were categorized as follows: increase in *p16^INK4a^* expression above the assay precision (an increase over 0.4); unchanged *p16^INK4a^* levels within the assay precision (between 0.4 and -0.4); and decrease in *p16^INK4a^* expression below the assay precision (below 0.4). Most patients had increases in *p16^INK4a^* expression, with percentages ranging from 49% for the TC group to 71% for the AC-TC group. The percentage of patients whose *p16^INK4a^* expression remained unchanged ranged from 12% in the AC-TC group to about 35% in both the AC-T and TC groups. Only a small percentage of patients (6%) in the AC-T had decreases in chemotherapy-induced *p16^INK4a^* expression, but this rose to 16%-18% in the other 3 chemotherapy groups. Therefore, although a choice of chemotherapy regimens may impact the magnitude of chemotherapy-induced increase in *p16^INK4a^* expression, an increase in *p16^INK4a^* expression is likely driven by the baseline *p16^INK4a^* expression and not chronological age or other patient and tumor characteristics.

## Discussion

We have previously shown that anthracycline-based chemotherapy in women with early stage breast cancer induced expression of cell senescence biomarkers in vivo (29). In the current study, we determined if chemotherapy regimens commonly used in patients with early stage breast cancers have differential effects on *p16^INK4a^* expression. We found that anthracycline-based therapies were statistically significantly more pro-aging than nonanthracycline-based therapies. Adjuvant anthracycline-based chemotherapy regimens save the lives of many patients and lower their risk of breast cancer relapse and death (33). However, potential toxicities such as myelodysplasia, acute leukemia (34,35) and cardiac toxicity (36) detract from the overall benefit. For many breast cancer patients, nonanthracycline-containing adjuvant chemotherapy regimens are associated with similar improvements in disease-free and overall survival as anthracycline regimens (37). Therefore, choosing a chemotherapy regimen that has a smaller impact on the chemotherapy-induced acceleration of aging may prove to be important to minimize chemotherapy-induced acceleration of morbidity and mortality.

Interestingly, although chemotherapy regimen type may have been an important driver of the magnitude of change in *p16^INK4a^* expression, baseline *p16^INK4a^* expression levels also played a statistically significant role. Patients with lower *p16^INK4a^* values at baseline experienced a larger chemotherapy-induced increase in *p16^INK4a^* expression than patients with higher baseline *p16^INK4a^* values, and that association remained statistically significant even after adjusting for chemotherapy regimen. One potential limitation of our study is a smaller sample size (between 17 and 53 patients in each regimen group). Larger studies will improve the quantification of age acceleration induced by different chemotherapy regimens. In addition, similar effects of chemotherapy and irradiation on telomere length have been reported (14-18). Also, measurement of additional biomarkers of aging that capture various molecular mechanisms of aging, such as an increase in inflammation (the measurement of the senescence-associated secretory phenotype) (10), epigenetic changes (19,20) (DNA methylation panels), and changes in metabolism, may deepen our understanding of processes affected by chemotherapy. Taken together, our results suggest that the choice of chemotherapy regimen, as well as the baseline “molecular age” of the patient, are important factors that determine the pro-aging capacity of chemotherapy regimens.

Our observation that patients with lower baseline *p16^INK4a^* expression experience a larger increase in *p16^INK4a^* expression is unexpected. One explanation is that there may be a threshold to the number of senescent cells a tissue or organism can tolerate. Whereas senescence protects from cellular damage-induced tumorigenesis, the accumulation of senescent cells is detrimental to tissue function and regenerative capacity because of an increase in inflammation and depletion of stem cells. In naturally aged mice, the number of senescent cells has been reported as 1%-3% of total tissue (38,39), suggesting that the maximum number of senescent cells is capped and tightly controlled by the immune system. We have previously found in a cohort of human donors that *p16^INK4a^* expression levels in a population tend to plateau, suggesting that there is a saturation threshold for the accumulation of senescent cells in humans as well (40). Our data in this study demonstrate that patients with lower baseline *p16^INK4a^* expression experienced a larger increase in *p16^INK4a^* levels during chemotherapy treatments than patients with higher baseline *p16^INK4a^* expression, consistent with the observation that *p16^INK4a^* expression may plateau later in life.

Much research remains to be done to characterize the correlation between *p16^INK4a^* expression levels and human health. Although a link between *p16^INK4a^* expression and mortality has been demonstrated in animal models (41-43), a definitive link between senescence levels and the clinical manifestation of aging and mortality in humans has not been established. A few studies have begun to establish the correlation between *p16^INK4a^* expression and predisposition to chemotherapy-induced toxicities and the development of frailty. For example, we demonstrated that high levels of *p16^INK4a^* correlated with chemotherapy-induced adverse events, such as severe (grade 3 and 4) fatigue (44). In childhood survivors, chemotherapy-induced accelerated aging and increased morbidity and mortality have been well established, and new data suggest that chemotherapy increased *p16^INK4a^* expression in a dose-dependent manner and increased *p16^INK4a^* expression correlated with increased frailty in this cohort (45). Studies are underway to establish the correlation between *p16^INK4a^* expression and multimorbidities and mortality in middle-aged adult cancer patients as well as young adult childhood cancer survivors.

In addition to chemotherapy, radiation is also known to promote cellular senescence (46-49). In murine models, whole body radiation is a major inducer of senescence and *p16^INK4a^* expression (50). However, the effect of radiation on changes in *p16^INK4a^* expression in women with early stage breast cancer is less clear. In women with early stage breast cancers, radiation is limited to the breast (in earlier stage patients) or the chest wall and regional nodes (in women treated with mastectomy and with larger tumors and/or positive axillary lymph nodes). Because of the targeted tissue volume treated, radiation in breast cancer patients is not likely to have the same effect as in murine models. Our previous studies in early stage breast cancer patients who received anthracycline-containing chemotherapy did not find radiation to be a statistically significant contributor to the chemotherapy-induced increase in *p16^INK4a^* expression, and in a second larger cohort of patients not treated with chemotherapy, radiation had no effect on *p16^INK4a^* expression (29). In the current study, 73% of patients received breast or locoregional radiation. We are unable to clearly define the role, if any, of radiation and *p16^INK4a^* expression because of statistical interaction between variables, but we are able to demonstrate that the inverse correlation between baseline *p16^INK4a^* levels and the magnitude of chemotherapy-induced *p16^INK4a^* increases remain even in women who did not receive radiation treatment. Larger studies in cohorts of women who receive radiation only or chemotherapy with radiation would be able to tease out individual contributions of baseline *p16^INK4a^* expression, chemotherapy regimen, and radiation on the *p16^INK4a^* increases with treatment.

Altogether, our findings suggest that the pro-aging effects of chemotherapy regimens are mediated by the combined effects of the type of chemotherapy regimen and the patient’s baseline *p16^INK4a^* level. In addition, our data suggest that anthracycline regimens, especially when used in younger patients, accelerate biologic age. Adjuvant anthracycline-based chemotherapy regimens save the lives of many patients and lower their risk of breast cancer relapse and death (33,51). However, recent studies show that adjuvant nonanthracycline regimens may be as effective as anthracycline-containing regimens for large numbers of early stage patients (37). Our data provide additional support for selection on nonanthracycline regimens in women with early stage breast cancer unless the projected benefit for anthracycline regimens is clearly associated with improved outcomes (for example, women with triple-negative breast cancer or those with hormone receptor–positive breast cancer with 4 or more positive nodes). For those patients where anthracycline regimens are preferred, the protective effects of lifestyle interventions such as exercise or pharmacologic agents (52,53) (eg, senolytics) to retard accelerated aging effects in patients receiving chemotherapy are areas of intense interest requiring further research.

## Funding

This work was supported by the National Cancer Institute (R01CA203023), Breast Cancer Research Foundation (New York, NY), Kay Yow Cancer Fund (Cary, NC), and University Cancer Research Fund (University of North Carolina, Chapel Hill, NC).

## Notes

**Role of the funder:** The funder had no role in the design of the study; the collection, analysis, and interpretation of the data; the writing of the manuscript; and the decision to submit the manuscript for publication.

**Disclosures:** The authors have no conflicts of interest to disclose.

**Author contributions:** SSS, NM, and HBM were involved in the conceptualization, methodology, data curation, data analysis, and writing. AMD was involved in the methodology, data curation, data analysis, and writing. KRH, CKA, LAC, ECD, TAJ, GGK, MSK, RER, JCS, and HBM contributed patient resources to this study. All authors contributed to the data analysis and writing.

**Acknowledgments:** We greatly appreciate the active support of the oncology clinicians and their research staff at multiple sites and, most importantly, the breast cancer patients participating in our studies. The study sites are Duke University Medical Center/Cancer Institute, Ohio State University Comprehensive Cancer Center, MD Anderson Cancer Center, UNC Rex Healthcare, and UNC Cancer Center. We also thank Tucker Brenizer, Erin O’Hare, Emily Bell, Chad Wagoner, Will Pulley, Nancy Burns, and Amy Garrett for their unwavering commitment to study implementation best practices. We are indebted to the pioneering research in biomarkers of aging by Dr Norman Sharpless and his initial involvement and encouragement of our investigations in women with breast cancer. We also appreciate the help of Lena Randhawa in the preparation of this manuscript.

**Prior presentation:** Preliminary results were presented at the American Society of Clinical Oncology 2017 Annual Meeting, June 3, 2017, Chicago, IL: Shachar SS, et al. (2017). Changes in p16INK4a (p16) expression, a biomarker of aging, in peripheral blood T cells (PBTC) in patients receiving anthracycline (A) vs non-anthracycline (NoA) chemotherapy (CRx) for early stage breast cancer (EBC). *J Clin Oncol.* 2017:35(15 suppl):10060-10060.

## Data Availability

The data underlying this article will be shared on reasonable request to the corresponding author.
